# Identification of Plants That Inhibit Lipid Droplet Formation in Liver Cells:* Rubus suavissimus* Leaf Extract Protects Mice from High-Fat Diet-Induced Fatty Liver by Directly Affecting Liver Cells

**DOI:** 10.1155/2016/4282758

**Published:** 2016-06-26

**Authors:** Tomohiro Takahashi, Wataru Sugawara, Yuya Takiguchi, Kento Takizawa, Ami Nakabayashi, Mitsuo Nakamura, Michiyo Nagano-Ito, Shinichi Ichikawa

**Affiliations:** Laboratory for Animal Cell Engineering, Niigata University of Pharmacy and Applied Life Sciences (NUPALS), 265-1 Higashijima, Akiha-ku, Niigata-shi, Niigata 956-8603, Japan

## Abstract

Fatty liver disease is a condition in which abnormally large numbers of lipid droplets accumulate in liver cells. Fatty liver disease induces inflammation under conditions of oxidative stress and may result in cancer. To identify plants that protect against fatty liver disease, we examined the inhibitory effects of plant extracts on lipid droplet formation in mouse hepatoma cells. A screen of 98 water extracts of plants revealed 4 extracts with inhibitory effects. One of these extracts,* Rubus suavissimus* S. Lee (Tien-cha or Chinese sweet tea) leaf extract, which showed strong inhibitory effects, was tested in a mouse fatty liver model. In these mouse experiments, intake of the plant extract significantly protected mice against fatty liver disease without affecting body weight gain. Our results suggest that RSE directly affects liver cells and protects them from fatty liver disease.

## 1. Introduction

Fatty liver disease (FLD) is a disease in which abnormally large numbers of lipid droplets, mainly composed of triglyceride (TG), accumulate in liver cells. FLD is typically caused by excessive caloric consumption or alcohol intake. Nonalcoholic fatty liver disease (NFLD) is FLD not caused by alcohol consumption and its prevalence is estimated to be 20–30% of the population in Western countries [1]. NAFLD can induce chronic inflammation (nonalcoholic steatohepatitis, NASH) in the presence of oxidative stress and may result in cancer due to cirrhosis. Recently, NAFLD has come to be considered a hepatic manifestation of metabolic syndrome [1, 2]. Although caloric restriction is the preferred treatment for NAFLD, this lifestyle modification is difficult for most people to maintain. Thus, herbs and foods with anti-NAFLD activity that can be consumed as part of daily meals or beverages represent an attractive alternative treatment for the prevention or cure of NAFLD.

Lipid droplets in liver cells are intracellular storage sites for neutral lipids mainly composed of TG [3]. In addition to TG, the lipid droplets contain various proteins involved in maintaining lipid structure and regulating lipid metabolism [4].

In the present study we report the identification of plant extracts that inhibit lipid droplet formation in liver cells* in vitro* and* in vivo*. These plant extracts may be useful for the prevention and treatment of NAFLD.

## 2. Materials and Methods

### 2.1. Reagents

Gallic acid monohydrate, ellagic acid dihydrate, rubusoside, olive oil (suitable for lipase assay), and the TG E-Test Wako kit were purchased from Wako (Tokyo, Japan). Most of dried plants including dried* Rubus suavissimus* leaves (Chinese sweet tea or Tien-cha) were purchased from Onshindo (Saga, Japan). Other reagents were of analytical grade.

### 2.2. Cells and Culture Conditions

The mouse hepatoma cell line Hepa 1–6 was obtained from RIKEN Cell Bank. The cells were grown in humidified 5% CO_2_ at 37°C in DMEM containing 10% fetal bovine serum.

### 2.3. Preparation of Plant and Food Extracts

Dried plants were ground with a Labo Milser ML-2 (Iwatani Corporation, Osaka, Japan) and 0.05 g of the resulting powder was extracted with 500 *μ*L water at 60°C for 1 h. After centrifugation at 15,300 ×g for 5 min, the supernatant was transferred to a new tube and used for screening. These extracts were considered to be 100% concentrated. RSE prepared as described above was used for all cell culture experiments.

### 2.4. Screening of Plants That Are Used as Foods or Herbal Medicines

Lipid droplets were formed in hepatoma cells using the methods of Fujimoto et al. [5] with modifications. We used Hepa 1–6 cells instead of HuH7 cells. In short, the cells (2 × 10^4^) were suspended in 100 *μ*L DMEM supplemented with 10% FCS and seeded in each well of a 96-well plate and incubated overnight. The next day, the medium was replaced with 100 *μ*L DMEM containing 10% FCS, 0.6 mM oleic acid conjugated with 0.12 mM defatted bovine serum albumin (BSA), and plant extract (5% of final concentration in the medium) and further incubated for 24 h. After incubation, the cells were washed with PBS and fixed with 3.7% formaldehyde for 10 min. The cells were then equilibrated with 60% isopropanol and treated with Oil Red O solution (300 mg Oil Red O in 100 mL isopropanol) to visualize oil droplets.

### 2.5. Lipid Analysis of Cultured Cells

Hepa 1–6 cells (2 × 10^6^) in 10 mL DMEM supplemented with 10% FCS were seeded in a 10 cm dish and incubated overnight. The next day, the medium was replaced with 10 mL DMEM containing 10% FCS and 0.6 mM oleic acid conjugated with 0.12 mM defatted bovine serum albumin (BSA) and the herb extracts to be tested and further incubated for 24 h. Total lipids were extracted from the cells with 2 : 1 (vol/vol) CHCl_3_/CH_3_OH and evaporated to dryness. The lipids were then redissolved in a small volume of 2 : 1 (vol/vol) CHCl_3_/CH_3_OH, and chromatographed on silica-gel 60 TLC plates (Merck, Darmstadt, Germany) in 80 : 20 : 1 (vol/vol/vol) CH_3_(CH_2_)_4_CH_3_/C_2_H_5_OC_2_H_5_/CH_3_COOH. Total lipids extracted from the number of cells corresponding to 150 *μ*g protein were used for the analyses. After separation of the lipids, the TLC plate was sprayed with cupric acetate reagent (8% phosphoric acid containing 3% cupric acetate) and lipids were visualized by heating at 130°C. For quantification of TG, TLC images were processed with ImageJ (Rasband, 1997–2005) software. TG content was quantified using olive oil as standard.

### 2.6. Cell Growth Assay

Cell growth assay was performed according to Kueng et al. [6]. In short, the cells (2 × 10^4^) were suspended in 100 *μ*L DMEM supplemented with 10% FCS and seeded in each well of a 96-well plate and incubated for 24 h. After incubation, the medium was replaced with 100 *μ*L DMEM containing 10% FCS, 0.6 mM oleic acid conjugated with 0.12 mM defatted bovine serum albumin (BSA), and plant extract and further incubated for 12 or 24 h. After incubation, the cells were fixed with 0.25% glutaraldehyde for 5 min. The cells were then washed with phosphate-buffered saline (PBS) and stained with 0.1% crystal violet for 15 min. After the staining, 0.1% crystal violet was removed and the cells were washed with tap water. After the washing, 100 *μ*L of 10% acetic acid was inoculated in each well and the resulting solution (33 *μ*L) was transferred to each well of a new 96-well plate. To this, 64 *μ*L of 10% acetic acid was added to make 100 *μ*L and mixed. Absorbance at 570 nm of each sample was measured by Microplate Reader Model 550 (Bio-Rad, Tokyo, Japan).

### 2.7. Protein Assay

Total protein was assayed using the BCA protein assay reagent kit according to the manufacturer's instructions (Pierce, IL, USA).

### 2.8. Animal Experiments

Animal experiments were performed according to international ethics standards and animal protocols were approved by the Niigata University of Pharmacy and Applied Life Sciences (Niigata Japan). The use of animals complied with the guidelines established by the Animal Care Committee of Niigata University of Pharmacy and Applied Life Sciences. A normal diet (CLEA Rodent Diet CE-2) and a high-fat diet containing 32% fat by weight (HFD 32) were purchased from CLEA Japan, Inc. (Tokyo, Japan). RSE used in animal experiments was prepared as follows. Dried* Rubus suavissimus* leaves (100 g) were extracted with 500 mL of water at 60°C for 1 h. After cooling, the mixture was filtered using filter paper (No. 2 ADVANTEC, Tokyo, Japan), and the resulting filtrate was used as RSE. Male C57BL6J mice were purchased from CLEA Japan, Inc. (Tokyo, Japan) and used throughout the experiments. Mice were housed in standard laboratory conditions (18–23°C, humidity 55–60%, 12 h light/dark cycle) for at least two weeks before each study. All mice were fed a normal diet and had free access to water for two weeks prior to the study. After two weeks of acclimation, the mice were divided into three groups (*n* = 6 per group): the normal diet, HFD, and HFD + RSE groups. The normal diet group was fed a normal diet and had free access to water. Both the HFD and HFD + RSE groups were fed HFD32. The HFD group had free access to water and the HFD + RSE had access to RSE instead of water. The mice were fed the normal diet without RSE or the HFD with or without RSE for one month. Body weight was measured weekly. The diets, water, and RSE were replaced every three days.

### 2.9. Quantitation of TG in Liver

Liver tissues were excised, rinsed with cold PBS, and homogenized in 3 volumes of water using a BioMasher II (Nippi, Tokyo, Japan). Total lipids were extracted from 280 *μ*L of homogenate with 500 *μ*L of 2 : 1 (vol/vol) CHCl_3_/CH_3_OH three times. The organic phase containing lipids was washed with 0.1 M KCl and evaporated to dryness. The lipids were redissolved in a small volume of 10% Triton-X100 and quantified using the TG E-Test Wako kit (Wako, Osaka, Japan). All data collected were analyzed using an unpaired *t*-test after one-way ANOVA testing.

### 2.10. Histology

Mouse liver samples were collected, fixed in 10% formalin buffered solution, and then embedded in OCT compound, frozen in liquid nitrogen, and sectioned (5 *μ*M thickness). The sections were then dried on glass slides, treated with 60% isopropanol for 1 min, and stained with Oil Red O solution at 37°C for 15 min. Sections were then treated with 60% isopropanol for 2 min and washed with water. Nuclei were counterstained with Mayer's hematoxylin solution.

## 3. Results

### 3.1. Identification of Plants That Inhibit Lipid Droplet Formation

**Figure 1:**
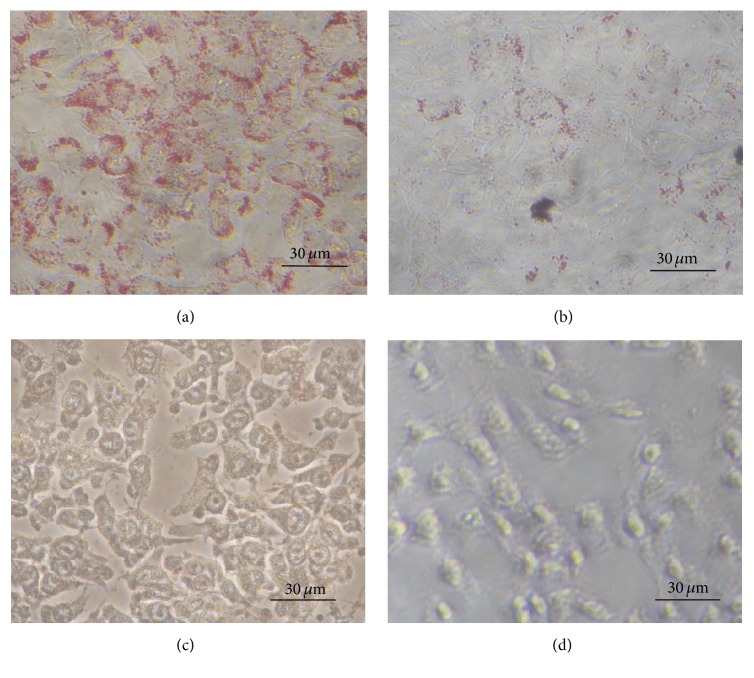
Inhibitory effects of plant extracts (5%) on lipid droplet formation in Hepa 1–6 cells treated with oleic acid. Dried plants were ground with a mill and 0.05 g of the resulting powder was extracted with 500 *μ*L of water at 60°C for 1 h. (a) No plant extract, (b) water extract of* Ginkgo* leaves (*Ginkgo biloba* L.), (c) water extract of* Hibiscus* flower (*Hibiscus sabdariffa* L.), and (d) water extract of* Platycodon* root (*Platycodon grandiflorum* A.). Details are described in Section 2.

**Figure 2:**
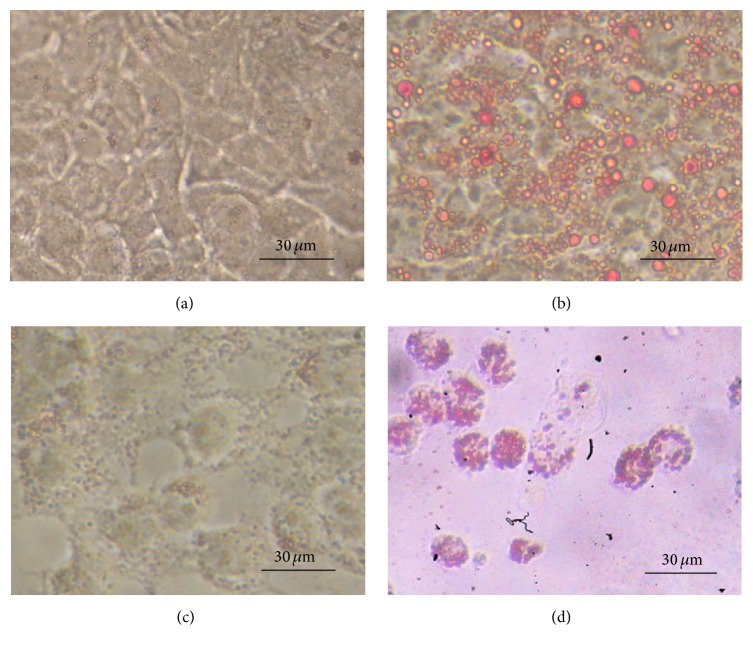
Lipid droplet formation in hepatoma cells treated with oleic acid and inhibition of lipid droplet formation by RSE. The mouse hepatoma cell line Hepa 1–6 was used for the experiments. Dried* Rubus suavissimus* leaves or* Nuphar japonicum* rhizome was ground with a mill and 0.05 g of the resulting powder was extracted with 500 *μ*L of water at 60°C for 1 h. The cells (20,000 cells/well) were seeded on a 96-well microtiter plate and incubated overnight. The next day, 0.6 mM oleic acid conjugated with BSA and the plant extract (RSE, 5% final or* Nuphar japonicum* rhizome extract, 1% final) was added and cells were further incubated for 24 h. After the incubation, the cells were fixed and oil droplets were visualized by Oil Red staining. Lipid droplets stained by Oil Red are seen as red particles. Details are described in Section 2. (a) Control, (b) 0.6 mM oleic acid, (c) 0.6 mM oleic acid and 5% RSE, and (d) 0.6 mM oleic acid and 1%* Nuphar japonicum* rhizome extract.

To screen plants for the ability to prevent NAFLD, we assessed the inhibitory activity of the extracts on lipid droplet formation induced by oleic acid in the mouse hepatoma cell line Hepa 1–6. We chose Hepa 1–6 cells because these cells contain a small number of lipid droplets when cultured in standard medium such as DMEM supplemented with 10% FCS (Figure 2(a)). In addition, this cell line has a fast growth rate and is very easy to handle. In the presence of oleic acid conjugated with bovine serum albumin, Hepa 1–6 cells form a large number of lipid droplets, similar to the lipid droplets seen in NAFLD livers (Figure 1(a)). Using this assay system, we screened 98 plants that are used as foods or herbal medicines. Four plant extracts tested positive for inhibition of lipid droplet formation induced by oleic acid. Those were extracts of* Ginkgo* leaves (*Ginkgo biloba* L.) (Figure 1(b)),* Hibiscus* flowers (*Hibiscus sabdariffa* L.) (Figure 1(c)),* Platycodon* roots (*Platycodon grandiflorum* A.) (Figure 1(d)), and* Rubus suavissimus* S. Lee leaves (Figure 2(c)).

**Figure 3:**
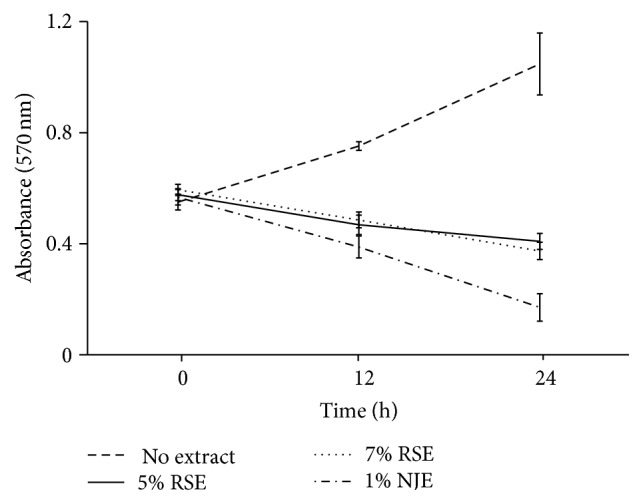
Effect of plant extracts on cell growth. Hepa 1–6 cells (2 × 10^4^) were suspended in 100 *μ*L DMEM supplemented with 10% FCS and seeded in each well of a 96-well plate and incubated for 24 h. After incubation, the medium was replaced with 100 *μ*L DMEM containing 10% FCS, 0.6 mM oleic acid conjugated with 0.12 mM defatted bovine serum albumin (BSA) and plant extract and further incubated for 12 or 24 h. After incubation, the cells were fixed with 0.25% glutaraldehyde for 5 min. The cells were then washed with phosphate-buffered saline (PBS) and stained with 0.1% crystal violet for 15 min. After the staining, 0.1% crystal violet was removed and the cells were washed with tap water. After the washing, 100 *μ*L of 10% acetic acid was inoculated in each well and mixed. A portion of the resulting solution (33 *μ*L) was transferred to each well of a new 96-well plate. To this, 64 *μ*L of 10% acetic acid was added and mixed. Absorbance (570 nm) of each sample was measured by Microplate Reader Model 550 (Bio-Rad, Tokyo, Japan). NJE:* Nuphar japonicum* rhizome extract. Data are means of five experiments presented with SD bars.

Among these, we focused on* Rubus suavissimus* S. Lee leaf extract (RSE) because a direct inhibitory effect on lipid droplet formation in liver cells had not previously been reported for this plant extract. As shown in Figure 2(c), treatment with RSE almost completely inhibited lipid droplet formation. To examine toxicity of RSE on Hepa 1–6 cells, we performed cell growth assay [6]. RSE-treated cells ceased proliferation and gradually reduced the number of the cells (Figure 3). Hepa 1–6 cells treated with 5% and 7% RSE showed toxicity to the same extent (Figure 3). Inhibition of lipid droplet formation was presumably not due to the toxicity because other extracts with greater toxicity did not suppress lipid droplet formation. For example,* Nuphar japonicum* rhizome extract showed strong toxicity at the concentration of 1% and the number of cells was reduced to 21% (Figure 3). However, even at this concentration, the lipid droplet formation was not inhibited (Figure 2(d)).

**Figure 4:**
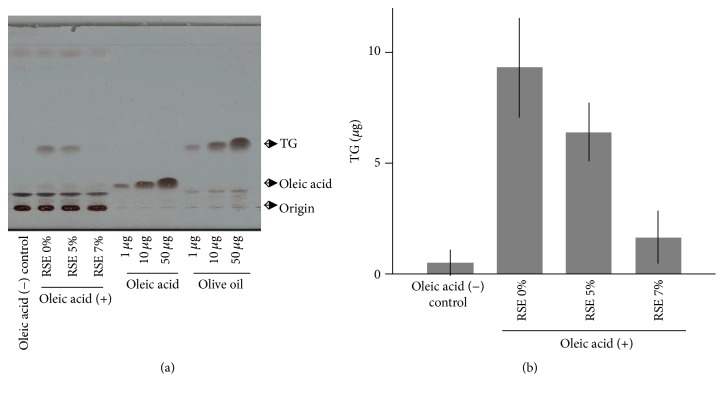
Effect of RSE on intracellular TG content in Hepa 1–6 cells. (a) TG content in RSE-treated Hepa 1–6 cells. Dried* Rubus suavissimus* leaves were ground with a mill and 0.05 g of the resulting powder was extracted with 500 *μ*L of water at 60°C for 1 h. This extract was considered to be 100% concentrated. TG in RSE-treated (RSE 5% and 7% final concentrations in the medium) and untreated Hepa 1–6 cells (RSE 0%) were analyzed by silica-gel TLC. The number of cells corresponding to 150 *μ*g protein was used for the extraction of total lipids and their analysis. After separation by TLC, lipids were visualized with cupric acetate reagent. TG (olive oil) and oleic acid standards of the indicated amounts are shown. (b) TG contents were quantitated after separation by TLC. Data are means of three independent experiments presented with SD bars. Details are explained in Section 2.

**Figure 5:**
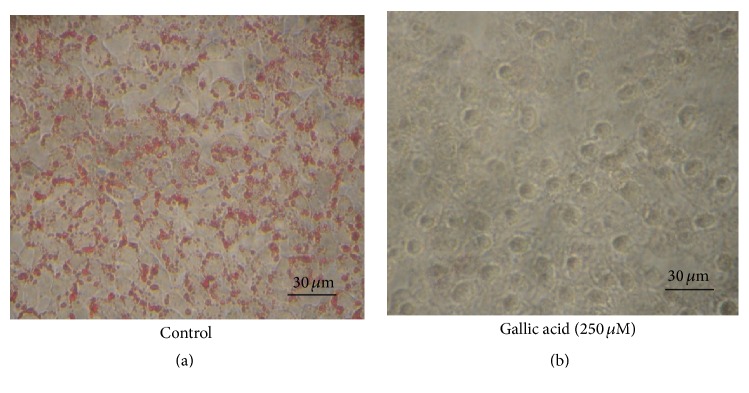
Effect of gallic acid on lipid droplet formation in Hepa 1–6 cells. The cells (20,000 cells/well) were seeded on a 96-well microtiter plate and incubated overnight. The next day, 0.6 mM oleic acid conjugated to BSA and gallic acid (250 *μ*M in the medium) was added and further incubated for 24 hr. After incubation, the cells were fixed and oil droplets were visualized by Oil Red O staining. Lipid droplets stained by Oil Red O are seen as red particles. Control: without added gallic acid.

We next analyzed TG content in oleic acid- and RSE-treated Hepa 1–6 cells. Cellular lipids were extracted, separated by silica-gel chromatography (TLC), and visualized with cupric acetate. Analysis of intracellular lipids revealed a dramatic increase in TG content in oleic acid-treated Hepa 1–6 cells, whereas no oleic acid was detected in the cells (Figure 4(a)). These results indicate that most of the oleic acid incorporated into Hepa 1–6 cells was converted to TG. As expected, a dose-dependent decrease in TG content was observed in cells treated with RSE (Figures 4(a) and 4(b)). As reported previously, the major small molecular components of RSE are gallic acid, ellagic acid, and rubusoside [7]. Thus, we next examined the effects of these compounds on Hepa 1–6 cells. Among the three compounds, only gallic acid (250 *μ*M) showed an inhibitory effect on lipid droplet formation, suggesting that it is an active component of RSE (Figure 5), whereas ellagic acid and rubusoside had no effect (data not shown).

**Figure 6:**
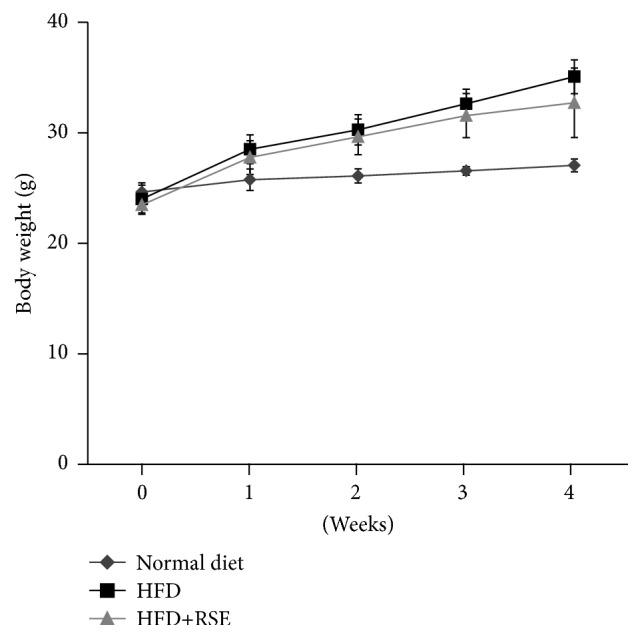
Body weight gain of mice fed HFD and treated with RSE. Body weight was measured weekly. Body weight gain was greater in HFD-fed mice than in those fed a normal diet. RSE extract had no significant effect on body weight gain in HFD-fed mice (*n* = 6 per group).

To determine if RSE is also effective* in vivo*, we performed animal experiments. A mouse NAFLD model was produced by feeding mice a high-fat diet (HFD) for one month with or without RSE to determine whether the extract had a protective effect against NAFLD. Average food intakes per mouse in the ND, HFD, and HFD + RSE groups were 7.3, 7.3, and 7.2 g per mouse per 3 days, respectively. Water or RSE intake was comparable among groups. Average water intakes in the ND and HFD groups were 12.6 mL and 9.2 mL per mouse per 3 days, respectively, and RSE intake in the HFD + RSE group was 10.4 mL per mouse per 3 days. We measured the body weights of all mice weekly. Body weight increased more in HFD-fed mice than in those fed a normal diet. RSE extract, however, had no significant effect on body weight gain in HFD-fed mice (Figure 6). The mice of the HFD + RSE group were apparently normal in appearance and behavior.

**Figure 7:**
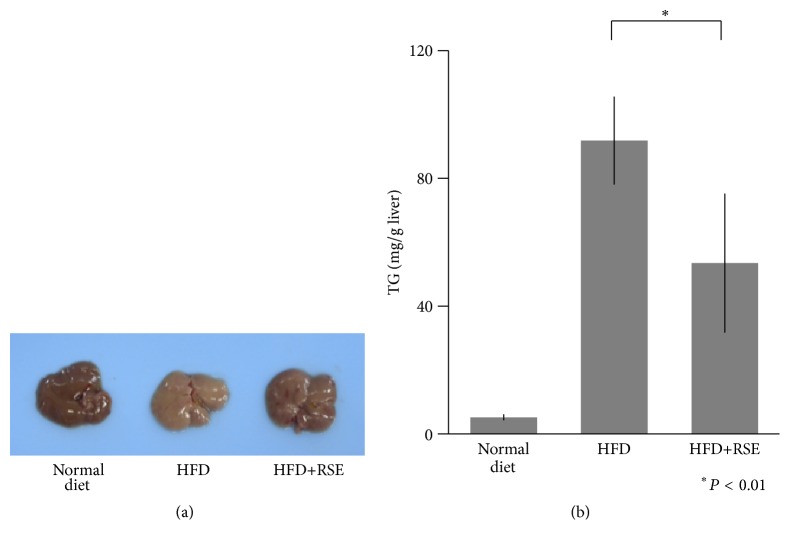
Effect of RSE on mouse fatty liver. (a) Mouse liver. As shown in the figure, the livers of normal diet-fed mice had a healthy red color, whereas those of HFD-fed mice had a white color due to TG accumulation. The livers of HFD-fed RSE-treated mice (HFD + RSE) partially retained their red color. (b) TG content in mouse liver. TG content in mouse liver was measured using the TG E-Test Wako kit. Data are the means of six experiments (*n* = 6 per group). Bar: SD.

**Figure 8:**
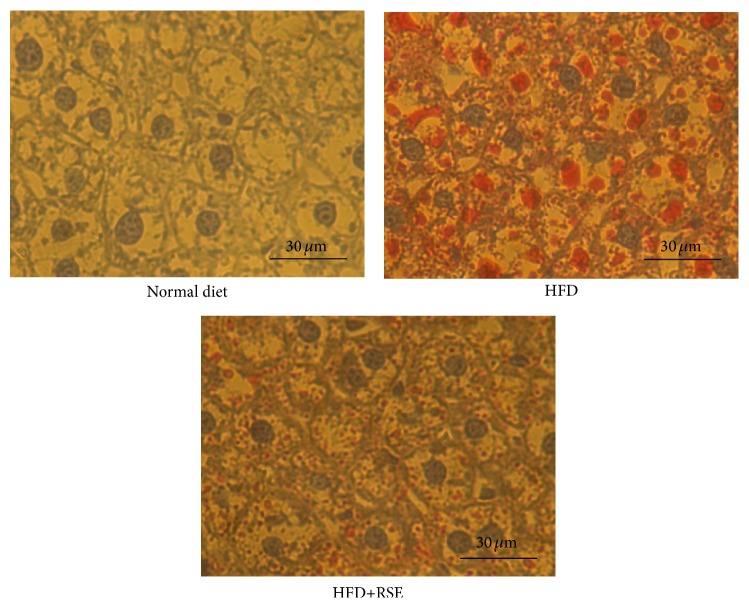
Histological detection of mouse NAFLD. Frozen sections of liver were prepared and after fixation, intracellular lipid droplets were stained red with Oil Red O. Nuclei were stained blue with hematoxylin staining to identify individual cells. Livers from a mouse fed a normal diet and mice fed a high-fat diet with (HFD + RSE) or without RSE (HFD) are shown. In the liver sections of HFD-fed mice, many red-stained lipid droplets were observed, whereas only a small number of lipid droplets were seen in the livers of mice fed a normal diet. The number and size of lipid droplets were much lower in the livers of RSE-treated mice (HFD + RSE).

The livers of the normal diet-fed mice were a healthy red color, whereas those of HFD-fed mice had a white color due to TG accumulation. The livers of HFD-fed and RSE-treated mice (HFD + RSE) partially retained a red color (Figure 7(a)). In the HFD-fed group, liver TG increased dramatically compared with that of controls. Treatment with RSE significantly decreased the accumulation of TG induced by HFD (Figure 7(b)). Histological examination of Oil Red O staining of cryosectioned liver tissue showed accumulation of lipid droplets in HFD-fed mouse liver whereas only a small number of lipid droplets were observed in the livers of normal diet-fed mice (Figure 8). As shown in the figure, lipid droplets in the livers of HFD-fed RSE-administered mice were greatly reduced, indicating a protective effect of RSE against fatty liver.

## 4. Discussion

A screen of 98 plant extracts identified 4 extracts with inhibitory effects on lipid droplet formation in a cell culture assay. Those were* Ginkgo* leaves (*Ginkgo biloba* L.),* Hibiscus* flowers (*Hibiscus sabdariffa* L.),* Platycodon* roots (*Platycodon grandiflorum* A.), and* Rubus suavissimus* S. Lee leaves. The protective effect of* Ginkgo* leaves against NAFLD had been reported by another laboratory in both animal [8, 9] and cell culture [9] models previously. In addition,* Platycodon* root extracts were shown to have protective effects on FLD in both nonalcoholic and alcoholic FLD animal models [10, 11]. The protective effect of* Hibiscus* flowers had also been reported in an animal model [12]. These results support the validity of our cell culture screening conditions. Since a direct protective effect against NAFLD had not been reported for RSE previously, we focused on this plant extract.* Rubus suavissimus* leaf (Tien-cha or Chinese sweet tea) is consumed as leaf tea in the Southwestern part of China [13] and in Japan it is used as a complementary alternative medicine to treat allergic rhinitis caused by pollen and household dust [14]. Koh et al. demonstrated that RSE or a mixture of the three dominant constituents of RSE, gallic acid, ellagic acid, and rubusoside mitigated obesity in high-fat diet-induced obese rats [7]. They also observed a reduction in TG content in the serum and organs, including the liver. In these experiments, however, the reduction of TG content could be considered a secondary effect of decreased obesity. Liu et al. showed that the gallic acid in RSE had antiangiogenic effects [15]. Koh et al. hypothesized that the antiangiogenic effects were responsible for the obesity-mitigating effects of RSE [7] since adipose tissue mass can be regulated by the vasculature [16]. In contrast to these results, in our experiments, RSE intake did not affect body weight in mice fed a high-fat diet, although it did prevent NAFLD. This difference may be due to differences in experimental conditions such as the animal species, route of administration, and RSE doses. Our data suggest that the protective effect of RSE against NAFLD in mice is mediated by a direct effect on liver cells for the following reasons. First, RSE did not affect body weight at the dose used in our study. Second, RSE was shown to inhibit lipid droplet formation in cultured cells similar to the mouse NAFLD model.

Gallic acid is a strong candidate for the effects of RSE on TG since its antisteatotic effect in rats and mice was reported previously [17, 18]. In fact, among the three dominant constituents, only gallic acid showed an inhibitory effect on lipid droplet formation in Hepa 1–6 cells. In addition, gallic acid is also present in* Hibiscus* flowers [12], which also had an inhibitory effect on lipid droplet formation (Figure 1). However, the effects of gallic acid in rats and mice could be indirect effects, because the reduction of body weights was observed in gallic acid treated groups [17, 18]. Other constituents in RSE might be responsible for the inhibition of the lipid droplet formation since gallic acid is more toxic to the cells at the effective concentration. In addition, the effective concentration of gallic acid is rather high. Chou et al. reported the content of gallic acid in dried* Rubus suavissimus* leaves [13] and it was 0.13% in average. According to their results, the concentration of gallic acid in 5% RSE is less than 38 *μ*M which is much lower than 250 *μ*M. It is also possible that gallic acid functions synergistically with other constituents in RSE.

## 5. Conclusion

In the present study we successfully identified a plant extract, RSE, which prevented NAFLD in cell culture and animal models. Our studies suggest that RSE, also known as Tien-cha and widely consumed in China and Japan as a beverage, shows promise for the prevention or treatment of NAFLD.

## Abbreviations

RSE: * Rubus suavissimus* leaf extract
TG: Triglyceride
TLC: Thin-layer chromatography
FLD: Fatty liver disease
NAFLD: Nonalcoholic fatty liver disease.
